# Monitoring and Evaluating Progress towards Universal Health Coverage in Ghana

**DOI:** 10.1371/journal.pmed.1001691

**Published:** 2014-09-22

**Authors:** Frank Nyonator, Anthony Ofosu, Mabel Segbafah, Selassi d'Almeida

**Affiliations:** 1 University of Health and Allied Sciences, Ho Volta Region, Ghana; 2 Ghana Health Service, Accra, Ghana; 3 World Health Organization Country Offices, Accra, Ghana

## Abstract

This paper is a country case study for the Universal Health Coverage Collection, organized by WHO. Mabel Segbafah and colleagues illustrate progress towards UHC and its monitoring and evaluation in Ghana.

*Please see later in the article for the Editors' Summary*

This paper is part of the PLOS Universal Health Coverage Collection. This is the summary of the Ghana country case study. The full paper is available as Supporting Information file Text S1.

## Background

Since independence in 1957, Ghana has explored sustainable ways of attaining the WHO-defined goal of Health for All. Following transition from a completely government-funded system post-independence, to a full cost recovery, out-of-pocket payment system, the government continues to pursue strategies to increase accessibility to health services.

## Universal Health Coverage: The Policy Context

Since 2003, the Government of Ghana has been implementing the National Health Insurance Scheme (NHIS) as the main strategy to progressively bridge financial access barriers and provide a social risk protection system [1]. The scheme complements the Community-based Health Planning and Services program—the national strategy to progressively reduce geographical access barriers to health services [2]. The concurrent strengthening of the District Health Systems will contribute to improving health outcomes.

## Monitoring and Evaluation for Universal Health Coverage

Ghana uses an elaborate system of periodic health sector reviews at district, regional, and national levels to assess progress on its sector-wide indicators outlined in its National Health Strategy, in concordance with the objectives of the four-year Health Sector Medium-Term Development Plan. This annual process, led by the ministry of health, organizes a comprehensive review system using a variety of tools such as the Holistic Assessment Tool, feeding into the Interagency Performance Review, and culminating in the National Health Summit.

Routine administrative health service data are enhanced by periodic population-based surveys; Demographic and Health Surveys (DHSs) (conducted every five years beginning in 1988) and Multiple-Indictor Cluster Surveys (MICS) (2006 and 2011) are used to evaluate health service performance emphasizing key health interventions and equity, and progress within wealth quintiles and other stratifiers. National Health Accounts (NHAs) exercises were conducted in 2005 and 2010.

## Progress towards Universal Health Coverage in Ghana

While child mortality trends alone are not a good indicator of universal health coverage (UHC) as many factors contribute to intervention coverage, mortality trends nonetheless offer a general measure to assess progress in reducing the gaps. Overall, under-five mortality rates have declined by about one-third since 1990 (MICS 2011 reports 82 deaths per 1,000 live births for 2007–2011, in comparison, Ghana DHS 1988 reports 155 per 1,000). Neonatal mortality rates declined much more slowly, with only a 5% reduction since 2003, while the gaps between the wealthiest and poorest households have widened in recent years.

**Figure 1:**
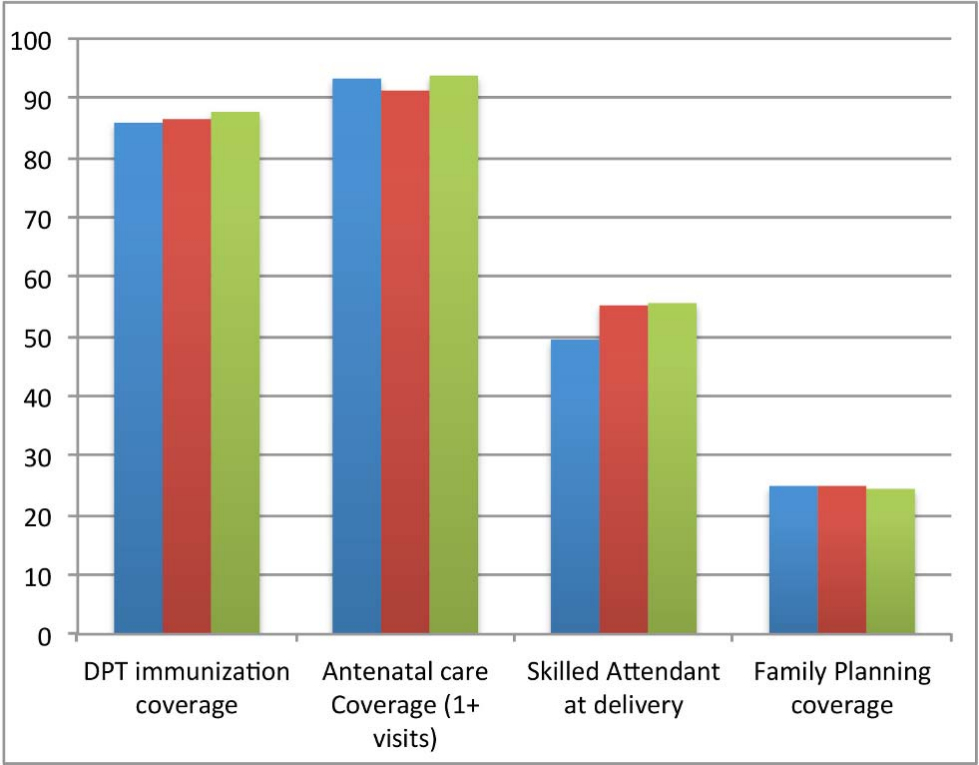
Trends in selected coverage indicators 2010–2012 (health facility data). Immunization rates are high, but skilled birth attendance and family planning coverage are still low and do not appear to be increasing. All coverages are in percentages (%). Blue, 2010; red, 2011; green, year 2012.

Coverage has been consistently high for United Nations Millennium Development Goal (MDG)-related interventions although increases have not been significant (Figure 1). Survey data indicate wide disparities in skilled attendant coverage by household wealth status. Only limited data are available on coverage for non-communicable disease interventions.

Inequalities in health worker distribution are significant, with little improvement in doctor-to-population ratio. There are 11-times fewer doctors per population in the Upper West Region in comparison to the Greater Accra Region, the latter representing approximately 50% of all Ghana's doctors [3].

Current national per capita expenditure on health is about 10% of the total national budget. Increments in health expenditure have not matched growth in the size of national income. Total health expenditure as a percentage of GDP fell from 6.4 to 3.3 from 2005 to 2010 [4],[5].

NHIS coverage in 2012 was 34% of the population compared to the target of 70%. Designed to be pro-poor, membership on NHIS favors the middle-wealth quintiles [6]. Out-of-pocket health expenditure remained the same at just under 30% of total health expenditure [4].

## Conclusions and Recommendations

To achieve UHC, increases in health sector resources should correspond to targeted investments in preventative, curative services and community-based care. The impediment to achieving UHC are two-fold: First, the poorly understood concept of cost containment in UHC and second, no mechanism for determining the basic package of services and how these reflect population needs over time.

In-country monitoring mechanisms and relevant evaluation tools are inadequate. There are significant gaps in quantifying equity and financial risk protection among different wealth quintiles, and in addressing the spread and control of non-communicable diseases and other chronic conditions.

Institutionalizing the National Health Accounts will provide a useful means for comparisons between investments and health outcomes.

With expected progress on expansion of pro-poor strategies, there is an urgent need to synergize both national strategies to achieve UHC and its desired impact. Expanding health insurance coverage to enhance quality care is key to the goal of UHC, and implementation within the current primary health care system will ensure that the lowest quintiles are not excluded.

National monitoring and evaluation frameworks should incorporate relevant global-level indicators that define and track country effective coverage for meaningful comparisons among countries of similar socio-economic and demographic characteristics.

## Supporting Information

Text S1 **The full country case study for Ghana.**
(DOCX)

## Abbreviations

NHIS: National Health Insurance Scheme
UHC: universal health coverage
