# Supplementary material for: Monitoring and Evaluating Progress towards Universal Health Coverage in Ghana
Source: PLoS Med. 2014 Sep 22;11(9):e1001691. doi: 10.1371/journal.pmed.1001691 (PMC4171378; doi:10.1371/journal.pmed.1001691)
Supplement: Text S1 — The full country case study for Ghana. (DOCX) [file pmed.1001691.s001.docx]

**Full Case Study: Monitoring and Evaluating Progress Towards Universal Health Coverage in Ghana**

Frank Nyonator^1^, Anthony Ofosu^2^, Mabel Segbafah^2^*, Selassi d’Almeida^3^

^1^ University of Health and Allied Sciences – Ho, Volta Region, Ghana

^2^Ghana Health Service - Accra, Ghana

^3^World Health Organization Country Offices, Ghana

**This paper is the full country case study to accompany the summary paper “Monitoring and Evaluating Progress Towards Universal Health Coverage in Ghana” that is part of the Universal Health Coverage Collection. Not commissioned; externally reviewed.**

*Corresponding author: Mabel Yayra Segbafah

Email: msegbafah@gmail.com

| **Abstract:** Ghana has been institutionalizing a set of coordinated mechanisms to measure and monitor progress in health coverage. These periodic assessments have over time shown significant country progress towards achieving a fifty-percent reduction in childhood morality rate by 2015 and a two-thirds reduction in maternal deaths within the same period. However, a lot more effort remains for Ghana to actually attain these Millennium Development Goals targets post 2015. The push for universal health coverage is now more important than ever and has necessitated the adoption of a systematic set of measurement and monitoring tools that will overtime depict effective country progress towards improved health outcomes, consolidating optimum health coverage. Ghana is working towards developing a system that is comparable with those in other countries of similar population structure and economic status particularly in Sub-Saharan Africa, to ensure more meaningful analysis of desired national health goals and outcomes. The purpose of this review is to determine whether Ghana as a country endorses universal health coverage and how well Ghana is positioned to achieve universal health coverage as well as which existing policies can or should be developed to achieve this. The review examines the systems operational for measuring universal health coverage in Ghana, strategies for identifying and addressing relevant gaps as well as which indicators will most appropriately assess progress towards universal health coverage. The review is contributing to the World Bank and World Health Organization (WHO) Measuring and Monitoring Universal Health Coverage PLOS collection  Nyonator, F. (2013) Universal Health Coverage: Extending Population Coverage in a Low Income Setting: The Experience of Ghana. (Unpublished) Abstract presentation at TICAD 5 August 2013.  Mensah J, Oppong JR and Schmidt CM. (2010) Ghana's National Health Insurance Scheme in the context of the health MDGs: an empirical evaluation using propensity score matching. Health Economics 19 S:95-106 |
| --- |

**Summary Points:**

1. Ghana has an elaborate range of indicator to measure and monitor its health outcomes, however, there is need for greater clarity on which indicators sufficiently measure true coverage and which ones measure impact or output

2. Policy-wise, success of Ghana achieving universal health coverage will depend to a large extent on Government leadership in promoting its key initiatives of community-based health care and services (CHPS), NHIS and Decentralization to increase focus on preventive and promotive health actions rather than large capital investments on hospital-based care in order to contain cost.

3. The move towards Universal Health Coverage will also depend on the private sector and technologies that will counteract the rising cost of health services, which remains a challenge to the public sector. Particularly because more non-poor access public sector service than the poor. Identifying which technologies to be provided within the system will and who decides is critical.

4. Much remains that is not well known and understood and requires further research. There is a need for further research on costs people are confronted with when accessing health services. There is need to promote research on incidence and prevalence of non-communicable diseases to assess their proportion on the burden of disease in Ghana.

**1. Background**

Utilizing local evidence-based research to strengthen health polices and improve outcomes is a major driver towards attaining Universal Health Coverage (UHC) (<http://www.who.int/health_financing/universal_coverage_definition/en/index.html>). Universal Health coverage as a concept relates to the population coverage of key health interventions. It also relates to the extent to which access to interventions is made possible and the financial risk protection that is provided to the population. The World Health Organization defines Universal Health Coverage as ‘Ensuring that all people can use the promotive, preventive, curative, rehabilitative and palliative health services they need, of sufficient quality to be effective, while also ensuring that the use of these services does not expose the user to financial hardship.’ Ghana has since independence in 1957 explored sustainable ways of attaining its goal of ‘Health for All.’ Successive Governments have over time demonstrated their commitment to this national goal by setting this as a health sector priority over successive years. This is further leveraged by the long standing Alma Ata Declaration in 1978 for universal access to Primary Health Care (PHC). The transition from a completely government-funded system that existed post-independence to a full-cost recovery out-of-pocket payment system in the early 1980s led to a reduction in utilization of health services [1]. Following these, Government has being pursuing a number of strategies to make health services more accessible and to increase utilization rate in the population [2]. Among the current strategies are the expansion of a community-level health delivery system and the institutionalisation of a social health insurance system funded primarily through tax. With these two interventions, Ghana is addressing the dimension of population coverage and providing financial risk protection for the population who are accessing services.

There is national consensus and concerted effort in institutionalising a National Health Insurance Scheme (NHIS) as main strategy that will progressively bridge financial barriers to access and provide a social risk protection system. Some studies show that there is progressive improvement in access to healthcare through the NHIS since its inception in 2003 [3,4]. The NHIS is to complement the Community-based Health Planning and Services (CHPS), the national strategy adopted in 1999 to reduce geographical access barriers to health services particularly in rural remote communities. CHPS employs a close-to-client approach [5] that enhances to the community health landscape by closing the physical gaps in access. The CHPS strategy is being augmented by the concurrent strengthening of the District Health Systems, in line with the Ouagadougou Declaration [6] (see Box S1) to improve national health outcomes [7,8].

| **Box S1: The Ouagadougou Declaration on Primary Health Care and Health Systems in Africa**  Achieving Better Health for Africa in the New Millennium was adopted during the International Conference on Primary Health Care and Health Systems in Africa, held in Ouagadougou, Burkina Faso, from 28 to 30 April 2008. Ghana has signed on to this to review past experiences on Primary Health Care (PHC) and redefine strategic directions for scaling up essential health interventions to achieve health-related MDGs using the PHC approach for strengthening health systems. Priority areas identified for strengthening were:   1. Leadership and Governance for Health; 2. Health Service Delivery; 3. Human Resources for Health; 4. Health Financing; 5. Health Information; 6. Health Technologies; 7. Community Ownership and Participation; 8. Partnerships for Health Development; and 9. Research for Health   These Priority areas now drive the Medium Term Health Strategic Plan for Ghana. |
| --- |

Positioning Ghana’s District Health System towards Universal Health Coverage

The District Health System has been structured and exists as a decentralised level within the health sector to plan and provide public health services, supervision and support to the lower levels – sub-district and community. While the health sector had decentralised its management and activities, this move had not been part of the national decentralisation schedule. In fact, the health sector had been on and off the national decentralisation schedule between 1988 and 2003, during which time the Ghana Health Service (GHS) was established through ACT 525 in 1996 [9]. Following a recent policy decision by the Parliamentary Cabinet in April 2012 the Health Sector will be reinstated unto the decentralisation schedule as a devolved department of the Metropolitan, Municipal and District Assemblies (MMDAs). An approved draft consolidated in the Local Government bill stipulates in Clause 63 (2) that the GHS at the district level will exist as a devolved Department of Health of the MMDAs [10]. This implies that the Local Government Service will now be responsible for provision of health services at the district level and below, with technical support from GHS (District Health System).

By also expanding the community-level health service delivery mechanism that affords communities to identify their own health needs and plan for them, and by institutionalising a comprehensive national social risk protection (health insurance) scheme, Ghana is set up towards universal health coverage post 2015. Further, the move to restructure the overall Governmental approach, will contribute to increasing the efficiency and effectiveness of the national health system.

The main objective of this review is to determine to the progress that Ghana has been making towards Universal Health Coverage using some proposed indictors, as a way of contributing to the global discussion on how best countries can measure their progress towards universal health coverage. The scope of the review included looking at the policies that have been adopted to ensure universal health coverage in Ghana, the national monitoring framework available for measuring progress towards UHC, a relook at the existing national indicators and those that can be used, the persisting gaps that need to be addressed to ensure better monitoring as well as the progress the country has made with regards to universal health coverage using the available indicators. A desk review of available literature and reports was done.

**2. Universal health coverage: the policy context**

For Ghana, the concept of UHC has been embraced and is couched in the country’s Health Sector Medium-Term Development Strategy document (1997 – 2001) [11]. The document elucidates the Government’s Developmental Agenda – “Towards Vision 2020,” which underpinned the Health Sector Reforms in Ghana to “Provide universal access to basic health services and improve the quality and efficiency of health services, as well as foster linkages with other sectors.” There are five strategic objectives (see Box S2), which provide the framework for monitoring and evaluating national progress towards universal health coverage. The health-financing mechanisms are also guided by laws that support rollout of financial protection.

| **Box S2: Five Strategic Health Sector Objectives that drove the Health Sector Reforms in Ghana**   1. Increased geographical and financial access 2. Better quality of care in all health facilities and during outreach 3. Improved efficiency in the health sector 4. Closer collaboration and partnership between Health Sector and communities, other sectors and private providers 5. Increased overall resources in the health sector equitable and efficiently distributed. |
| --- |

The Ghana Health Sector has developed and is implementing policies that would facilitate the acceleration and expansion of health services and service delivery points, as well as measures to increase relevant human resources for health, including trained community-level health workers. There is continuous strengthening of the health system using the health systems pathway built on the Health Systems Building Blocks (Figure S1), that is aligned to the nine priority areas of the Ouagadougou Declaration on Primary Health Care and Health Systems. This model, which was adapted from a paper on Community Based Health Planning and Services in 2005 [12] demonstrates the early conceptualization of universal health coverage in Ghana. It was geared towards improving access to service using the Community-based Health Planning Services (CHPS) strategy. With the subsequent introduction of the National Health insurance Scheme that provided financial risk protection, the current national concept for universal health coverage in Ghana is as shown in Figure S3.

**Figure S1:** Ghana Pathway To Universal Health Coverage (UHC). Data Source: Modified from WHO’s Health Systems to Improve Health Outcomes [12]

| ***Inputs*** |  | ***Outputs*** |  | ***Coverage*** |  | ***Impact*** |
| --- | --- | --- | --- | --- | --- | --- |
| *Financing*  *Health workforce*  *Medicines etc.* |  | *Service delivery*  *Pooled financial risks* |  | *Intervention coverage*  *Risk factor reduction*  *Coverage with financial risk protection* |  | *Better health*  *Improved household wealth and protection against poverty*  *Responsiveness* |

**Figure S3.**

Ghana’s current per capita on health is about 10% of the total national budget. No government so far has achieved the Abuja target of 15% of recurrent budget on health spending. Interestingly to the contrary, governments’ over successive years have reduced the relative budgetary allocation to health, despite progressive improvements in the national fiscal space. (Table S1) [13,14]

| **National Health Accounts** | **2005 (US$M)** | **Percentage of THE** | **2010 (US$M)** | **Percentage of THE** | **Notes** |
| --- | --- | --- | --- | --- | --- |
| **Total Health Expenditure (THE)** | 680.5 |  | 964.6 |  | Over 42 percent increment over 5 years |
| **THE as a percentage of Gross Domestic Product** | 6.4 % |  | 3.2% |  | The increase in the size of the economy did not match the expansion in health expenditure |
| **Public funds from the Government of Ghana** | 180.6 | 26.5% | 384.9 | 39.9% | Over 113 percent increment over 5 years |
| **Public funds from the National Health Insurance Fund** | 20.7 | 3.1% | 277.9 | 28.8% | Over 1000 percent increment over 5 years |
| **Cost of general health administration and insurance** | 211.3 | 31.1% | 428.1 | 44.3% | Over a 100 per cent increase |
| **Expenditure on provision and administration of public health programme** | 228.4 | 33.5% | 463.5 | 48.1% | 79.63 per cent reduction |
| **Direct out-of-pocket payments** | 113.6 | 16.7% | 112.6 | 11.6% | Fell within the five year period |
| **Expenditure at the hospitals** | 146.2 | 21.4% | 376.6 | 39.1% | Over 100 per cent increase between 2005 and 2010 |
| **Per Capita Expenditure (in US$)** | 32.3 | - | 39.1 | - | Per capita expenditure on health has been increasing |

**Table S1:** National Health Accounts 2005[13] and 2010[14]

A rebasing of Ghana’s GDP in 2010 saw over 200% increment in nominal dollar terms. However, the increment in health expenditure has not matched growth in the size of the national income, thereby making total health expenditure as a percentage of GDP to fall from 6.4 percent in 2005 [13] to 3.2 percent in 2010 [14] (This is a phenomenon that has also been documented in other developing countries that have experienced growth in the economy over the past years). While the projections of the Global Health Observatory of the World Health Organization show a gradual fall in government’s health expenditure over 2005 to 2010 (Table S2), the National Health Accounts carried out in 2005 and 2010 depicted a sharp fall. In comparison to other countries with similar income, Ghana’s per capita spending on health is relatively low with relatively high out-of-pocket spending (Figure S2). It becomes more evident that if Ghana is to achieve universal health coverage, there has to be corresponding increase in resource flow to the health sector and targeted investments in prevention and promotion, and community-based care rather than hospital-based care in order to contain costs.

**
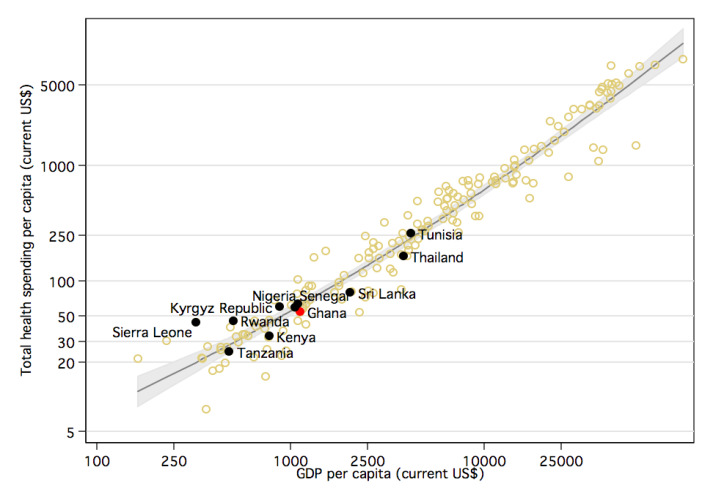
**

**(a)**

**
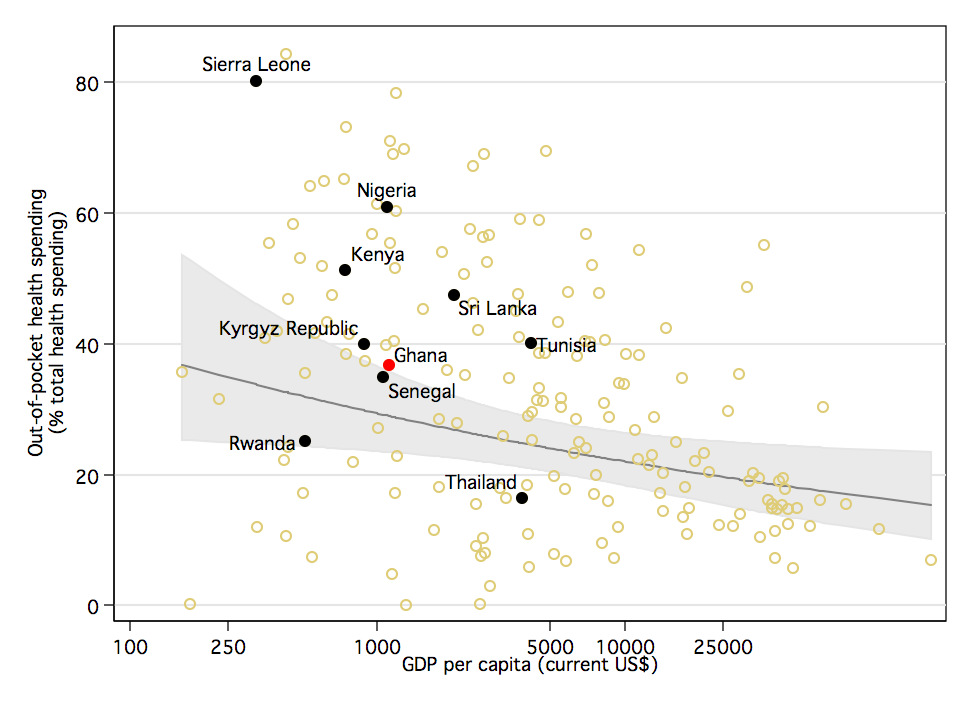
**

**(b)**

**Figure S2:** Per capita health spending compared to countries with similar incomes, 2009

1. Total Health Spending
2. Out-of-pocket Health Spending

Data source: Source: Saleh K (2012) WORLD BANK STUDY: A Health Sector in Transition to Universal Coverage in Ghana. Available: https://openknowledge.worldbank.org. Accessed 13/11/2013

|  | | | | | | | |
| --- | --- | --- | --- | --- | --- | --- | --- |
| **Indicator** | **2005** | **2006** | **2007** | **2008** | **2009** | **2010** | **2011** |
| Population (in thousands) total | 21,384 | 21,948 | 22,526 | 23,110 | 23,692 | 24,263 | 24,821 |
| GDP per capita (2008 PPP US$) (http://hdr.undp.org) | 1,292 | 1,345 | 1,392 | 1,463 | 1,494 | 1,533 |  |
| Total expenditure on health as a percentage of gross domestic product | 7.02 | 5.36 | 6.01 | 5.59 | 4.97 | 5.2 | 4.78 |
| General government expenditure on health as a percentage of total expenditure on health | 66.35 | 57.22 | 62.36 | 58.04 | 56.53 | 58.22 | 56.09 |
| Per capita total expenditure on health at average exchange rate (US$) | 34.81 | 47.16 | 65.55 | 68.52 | 54.19 | 68.53 | 75.02 |
| Private prepaid plans as a percentage of private expenditure on health | 10.99 | 6.67 | 6.3 | 6.34 | 6.25 | 6.26 | 6.22 |
| Per capita government expenditure on health at average exchange rate (US$) | 23.1 | 26.98 | 40.87 | 39.77 | 30.64 | 39.89 | 42.08 |
| Per capita total expenditure on health (PPP int. $) | 84.79 | 67.69 | 83.27 | 83.73 | 76.42 | 85.25 | 90.01 |
| Per capita government expenditure on health (PPP int. $) | 56.26 | 38.74 | 51.93 | 48.59 | 43.2 | 49.63 | 50.49 |
| General government expenditure on health as a percentage of total government expenditure | 15.25 | 14.8 | 15.44 | 12.22 | 12.47 | 12.08 | 11.87 |
| External resources for health as a percentage of total expenditure on health | 23.59 | 17.87 | 13 | 10.97 | 13.52 | 16.47 | 14.16 |
| Social security expenditure on health as a percentage of general government expenditure on health | 21.74 | 10.6 | 33.61 | 26.21 | 27.37 | 25.23 | 23.73 |
| Out-of-pocket expenditure as a percentage of private expenditure on health | 63.73 | 67.03 | 67.19 | 67.59 | 66.65 | 66.71 | 66.29 |
| Private expenditure on health as a percentage of total expenditure on health | 33.65 | 42.78 | 37.64 | 41.96 | 43.47 | 41.78 | 43.91 |

Table S2: Health Expenditure in Ghana (2005-2011)

Data Source: Global Health Observatory of the World Health Organization, 25 September 2013 http://apps.who.int/gho/indicatorregistry/App_Main/view_indicator.aspx? iid=10.

Creating and Promoting Equity in Access to Health Care Services

Efforts to promote equity in access to health services are a priority on the health sector agenda. National wide rollout of the CHPS (see Box S3) represents the health sector’s direct contribution to the National Poverty-Alleviation Policies. CHPS implementation is equity-focused first targeting areas of highest need – remote and hard-to-reach areas and evolving to cover urban slums. The CHPS concept is strongly supported by Government and its development partners in health and serves as the backbone of community-level interventions. The CHPS close-to-client approach has been found to be the most effective in significantly and progressively increasing the number of health service encounters and improving quality of family planning and maternal health services with concurrent significant reduction in childhood immunization dropout rates [15, 16]. CHPS enhances communication and integration among local authority, social groups and other decision-making systems in communities and consolidates their community-level actions to improve health services [17].

| **Box S3: Community Based Health planning and Services (CHPS) Strategy**  In the CHPS Strategy, the trained health worker is relocated from static health centres and integrated into communities to provide a basic but essential health service package and information. By creating spaces to carry out these community level health services, the health system in Ghana has created a third tier of health service delivery called the CHPS zone, which is a demarcated area within which these essential services are carried out to a defined population of about 5000 people. CHPS zones are planned and evolve around health centres that serve as the next level in the referral system. Thus, their network is towards reducing travel distance and time (improving geographic access) to seek health services and shortening waiting time at larger health facilities.[9] |
| --- |

The NHIS in Ghana, is set up as a social protection mechanism and designed to complement the CHPS strategy [9]. NHIS is essentially an enhancement of the district-level mutual health insurance scheme (DMHIS) [18]. NHIS serves to re-establish the relationship between the health system and household, which had been strained by the cash-and-carry system introduced in the 1980s. The NHIS partners with the Livelihood Empowerment Against Poverty (LEAP) programme and other community-level actions to identify and enable poorer and most vulnerable individuals and households access health care services and good nutrition.

Although the two strategies are equity-focused, adapting both strategies to the country’s changing economic climate and health needs of Ghanaians has been most challenging. A key challenge is “how to establish an effective social protection scheme that reduces catastrophic out-of-pocket expenditure.” While the onus rests in providing risk protection, it seems that those responsible for providing risk protection do not themselves seem to fully comprehend that the whole financial arrangement is to ensure universal health coverage. While the concept of cost containment through the NHIS does not seem to be fully understood, the absence of a mechanism for determining the elements of a basic package of services and how this evolves over time impedes progress of current efforts. It is however becoming clearer that social risk protection must aim to complement the efforts of closing geographical access barriers.

For instance, routine health service data shows that where individuals have an active health insurance there is a progressive increase in outpatient attendance. In 2011, the proportion of insured clients accessing OPD services increased from 56% in 2010 to 82%, with CHPS contributing approximately 5% to the total OPD attendance. In 2012, while the proportion of OPD attendance by insured clients dipped to 80% (The dip is attributable to the capitation pilot in Ashanti Region), CHPS contribution to total OPD attendance remained progressive, increasing to 6% [19]. Thus, as CHPS coverage is expanding, NHIS coverage and CHPS contribution to outpatient attendance should remain progressive.

Despite this impact, the current inability of the scheme to reimburse service providers without extended waiting times has caused a perceived provider preference for uninsured clients over insured clients, as uninsured clients will pay-out-of-pocket and increase providers’ revenue; the other side of which is that clients prefer to seek health services at the tertiary level and may be willing to pay out-of-pocket to the detriment of the lower level primary health care facilities, thus challenging efforts towards strengthening the primary health care and referral systems [20]. This depicts the peculiar challenge to cost containment as active membership of the scheme grows.

In 2012 the population targeted to be active NHIS cardholders was 70%. The achieved coverage was 34% - a little less than half of the expected [21]. Membership on the NHIS although designed to be pro-poor has shown in many research studies to be favouring the middle third and fourth wealth quintiles over the poorest [22, 23].

| **Box S4: Financial protection laws and policies in Ghana**  N.B.: The other policies and instruments mentioned indicate that Ghana has made various provisions for financial protection prior to the commencement of the NHIS and LEAP which are the main ones currently being implemented.   1. Social Security Act – 1965// Provident Fund Scheme for old age, invalidity, death-survivor benefits. 2. Social Security Law – 1991// Conversion of the provident fund scheme to social security and national insurance trust (SSNIT). 3. Ghana poverty reduction strategy – 2002 – 2005// Focused on achieving the MDGs. 4. National Health Insurance Scheme – 2003 // Contribution scheme for health insurance 5. Growth and Poverty Reduction Strategy II – 2006 – 2009// Achieving middle-income status by 2015. 6. National Social Protection Strategy – 2007 // Introduction of Livelihood empowerment against poverty (LEAP) and other strategies.   LEAP – 2008// Target poorest quintiles; provide social cash transfers and free health insurance (NHIS) |
| --- |

**3. Monitoring and evaluation for UHC**

Ghana uses routine administrative health service data to monitor and report on the annual health sector performance. This is complemented by the two periodic population-based surveys – the Demographic and Health Survey (DHS) and Multiple-Indictor Cluster Survey (MICS) to evaluate health service performance and utilisation coverage. This is further complemented by other household surveys such as the Living Standards Survey (LSS) and the Demographic Surveillance System (DSS).

Over time, the GHS has made huge investments to improve the routine administrative health data collection system and gradually shifting from a well-established yet enormous paper-based and parallel reporting system to an electronic web-based integrated system, which allows the sector-wide indicators to easily be monitored. This system is operational as the District Health Information Management System 2 (DHIMS2) that is built on the dhis2 platform [24]. The system is designed to consolidate routine service data collated from sub-district and district levels and is complemented by Standard Operation Procedures and a Monitoring and Evaluation Plan to ensure accuracy and consistency in definitions and measurement of indicators.

Within the broader Health Sector there are standard periodic reviews at District, Regional and National levels that report on performance of sector-wide indicators developed along the five strategic objectives and include indicators that potentially measure UHC. Under the Ministry of Health these reviews feed into the Interagency Performance Reviews and culminate into the Health Summit. The annual assessment of the health sector performance is achieved using a variety of tools such as the Holistic Assessment Tool [25]. Equity is a major focus of the Health Sector Medium-Term Development Plan (HSMTDP 2010-2013). Equity of the services rendered is mostly monitored through Demographic and Health Surveys and the Multi-indictor cluster surveys. The Health Sector Objective one focuses entirely on achieving equity in health (Table S3, end of file). The monitoring and evaluation framework currently being used in Ghana is as shown in Table S3 (end of file). It is ordered around the five strategic objectives of the Health Sector Medium Term Development Plan. (HSMTDP 2010- 2013) with key indicators to monitor progress under each strategic objective. There are gaps in the current monitoring framework that needs to be addressed to ensure a better measurement of progress towards universal health coverage by Ghana.

**4. Progress towards UHC in Ghana**

The indicators chosen to measure UHC were based on their availability in the monitoring framework, their high epidemiological and public health significance. From the framework presented in Figure S1, the indicators that are useful in our context to monitor progress towards achieving UHC are outcome indicators that measures increased access to and use of services, improved quality of services and improved efficiency. Indicators measuring enhanced service delivery are also useful to measure UHC. Finally from Figure S3, as universal health care is to result in better health outcomes impact indicators mainly mortality indicators are also very useful to be used to measure progress towards achieving UHC.

Pooling Financial Resources

Increased Access to and use of services

Access to Nearest Facility (DHS1993 compared to DHS 2008)[26, 27]

The indicator to measure this is “Respondents who admitted that Distance to Health Facility was a barrier to accessing health care.” Among the rural population this has improved from 47% (DHS 2003) to 34% (DHS 2008). Comparing among wealth quintiles, among the lowest quintile there has been improvement from 60 % (DHS 2003) to 50% (DHS 2008). The Universal Health Coverage policy impact that can be assessed is the increase the percentage of functional CHPS zones against the demarcated zones or expected zones and the population being served by CHPS. Access to services to prevent, manage and provide rehabilitative services for non-communicable diseases should be a Universal Health coverage priority in the future, with regards to equity; the performance of the country has not been encouraging.

Assessment of access to emergency neonatal and obstetric care (EmONC Needs Assessment 2010) has found Inequitable distribution of skilled human resources (especially midwives), inequitable and insufficient distribution of properly equipped facilities, Poor quality and comprehensiveness of services, even in wealthier populations. This leads to low uptake of services by those in the lower wealth quintiles and some geographical areas. The gaps are widening between the rich and the poor for most of the indicators [28]. Table S4 shows the situation for equity index for under-five mortality using wealth quintiles in the DHS and MICS.

| **Indicator** | **Target** | **Performance** | **Source** |
| --- | --- | --- | --- |
| Equity: Under five mortality ratio comparing the fifth wealth quintile to the first wealth quintile | 1:1.5 | 1:2.04 | MICS |
| Equity: Geography - Services Supervised deliveries comparing the best performing region to the worst performing region | 1:1.70 | 1:1.53 | GHS |
| Equity: Geography - Nurse: population ratio comparing the best performing region to the worst performing region | 1:1.95 | 1:1.75 | MOH |
| Doctor: population ratio | 1:9,700 | 1:10,452 | MOH |
| Nurse: population ratio | 1:900 | 1:1,251 | MOH |

**Table S4**: Health Sector Performances on Equity 2012

With twice as many under-fives dying per 1,000 live births among the poorest in comparison to least poor, the under-five mortality inequality gap is widening. While survival of children in the least poor households seems to be on track to meet the MDG target, children from other wealth quintiles and especially the poorest are faring much worse. The same widening inequity gap is observed in mortality rates of infants and neonates.

There has been no significant reduction of infant mortality observed since 2003, with only 5% reduction in neonatal mortality among the poorest children. However, there has been 35% and 43% reduction in infant and neonatal mortality rates, respectively among the least poor children in the same period[29, 30]. The reasons for these poor outcomes can be attributable in part to the inequitable distribution of health workforce and to other socio-economic determinants, knowledge and practices.

Improvement in the doctor to population ratio has not been significant over successive years. There are eleven times less doctors per population in comparing the Upper West Region to Greater Accra Region. While equitable distribution of doctors remains a major challenge to the health sector, Greater Accra Region continues to attract and retain the highest number of doctors per capita with one doctor per 3,540 inhabitants. Fifty percent of all Ghana’s doctors are in Greater Accra Region and another twenty percent are in Ashanti Region [24]. In assessing equity although wealth quintile segregation is useful way to assess equity, it is only available through survey data. Regional classification although affected by some confounders is collected routinely and hence can be assessed and compared on a year-to-year basis. In Ghana the north and south socio-economic divide makes equity analysis by regions very relevant.

Impact Indicators

Although mortality is not a good measure for universal health coverage as there are other factors involved. Our conceptual framework on achieving universal health coverage selected mortality indicators including neonatal mortality, infant mortality and under-five mortality provide a useful although biased proxy for measuring and assessing impact of preventive, promotive and curative services.

For instance Figure S5 shows a progressive decline in neonatal and infant although not as significant as observed for under-five mortality rates over the last 25 years. Neonatal mortality rates seem to have stagnated, due in part to the low access and utilisation of skilled assistance at birth (Figure S4). On the other hand increasing vaccination coverage has had a significant impact on improving under-five survival rates (Table S5).

**Figure S4:** 10-year trend of Family Planning ANC and Skilled Delivery Coverage

(2003 – 2013)

**Figure S5:** Under-five, Infant Mortality and Neonatal mortality.

Data Source: 2011 National MICS Presentation – UNICEF Ghana at Nov 2012 MOH Health Summit

| Indicator | **Target** | **2007** | **2008** | **2009** | **2010** | **2011** | **2012** |
| --- | --- | --- | --- | --- | --- | --- | --- |
| **Penta 3 Coverage** | ≥90% | 87.8 | 86.6 | 89.3 | 87 | 87 | 88 |
| **OPV3 Coverage** | ≥90% | 87.6 | 86.1 | 88.7 | 87 | 87 | 87 |
| **BCG Coverage** | ≥90% | 100 | 100 | 100 | 102 | 105 | 104 |
| **YF Vaccination Coverage** | ≥90% | 88.1 | 86.1 | 88.8 | 88 | 87 | 88 |
| **Measles Vaccination Coverage** | ≥90% | 88.6 | 86.5 | 89.1 | 88 | 88 | 89 |
| **TT2 Vaccination Coverage** | ≥90% | 70.1 | 76.3 | 78.6 | 76 | 76 | 74 |
| **Proportion (%) Children fully immunized by age 1 year (DHS/MICS)** | ≥80% |  | 91 |  |  | 91 |  |
| **BCG/Measles Drop out rate** | ≤1% | 11.9 | 13.9 | 11.2 | 14 | 16 | 11 |

**Table S5**: Immunization Coverage of BCG, OPV3, Yellow Fever, Measles and TT2

Life expectancy has improved and continues to be progressive, increasing nearly ten years; from 54 years in 1988 to 63 years in 2010 [31]. This corresponds to the projected increase in life expectancy from 58 years (2008), to 65 years (2010) and to 70 years by 2020 [32, 33]. Ghana is among the African countries named to have achieved the MDG 1 target of halving poverty by 2015. There has been progressive improvement in the poverty headcount ratio; national poverty line from 51% (of population) in1992 down to 28% (of population) in 2006 [34]. There is an increase in non-communicable disease. Routine data on this condition is not reliable since most of those with these conditions are not seen at the health facilities from the SAGE study conducted in 2008 [35].

The analysis above suggests that whilst Ghana has favourable in-country monitoring mechanisms and the relevant tools to measure its progress towards achieving its targets and goals for attaining universal health coverage, there are still some significant gaps particularly in the areas of measuring equity and financial risk protection, particularly among the different wealth quintiles. There is a need for adapting more suitable outcome measures on the poorest quintiles to monitoring the risk of catastrophic out-of-pocket payments that will inevitably plunge populations and households in the third and fourth quintiles into poverty. Ghana has made significant progress in tracking its communicable diseases overtime but there is the a need to capture progress on spread and control of non-communicable diseases as well as chronic conditions in the population particularly as Ghana’s population matures and its adult age population continues to increase.

Whereas the Ghana National health indicator review process may be elaborate, there is also the need to bring this into the global context. This can be done through a number of ways which includes institutionalising the National Health Accounts to compare investments more rigorously with health outcomes; adding/ including variables on non-communicable diseases particularly hypertension, diabetes and cancers into already existing national data collection systems such as the Demographic and Health Surveys, Ghana Living Standards Surveys and Multiple Indicator Cluster Surveys. Ghana must also constantly look for ways to incorporate and refine the quality of effective coverage indicators into the existing national as well as routine monitoring and evaluation frameworks. In addition these monitoring and evaluation frameworks must incorporate relevant global indicators to make meaningful comparisons among countries of similar socio-economic and demographic characteristics.

The implications of these are varied but will ultimate create a more robust country system for monitoring progress in Ghana’s selected sector-wide indicators as well as a more comprehensive framework for tracking country efforts towards the desired goal of universal health coverage.

**5. Conclusions and recommendations**

In conclusion, although individual countries may have peculiar disease profiles and burden of diseases therefore would define their peculiar health goals these must be meaning in providing a platform for comparison on the global drive towards universal health coverage, particularly because as our globe becomes ever smaller we will be ultimately responsible for creating systems that should address nearly all common health problems. Clearly, in Ghana, there is so much that is not well known at the country level and further research is needed to monitor and measure progress towards universal health coverage. The indicators currently being used do not accurately monitor progress in financial risk protection. In the way forward, there will be the need to adopt some global indicators that will help the country to monitor progress in these dimensions of universal health coverage.

| **Box S5: Recommendations**  1. There will be the need to add some global indicators on financial risk protection to the monitoring framework for UHC.  2. There will be the need for further research on the progress made towards achieving Universal Health Coverage. |
| --- |

**References**

1. Nyonator F and Kutzin J. (1999) 'Health for some? The effects of user fees in the Volta region of Ghana. Health Policy and Planning 14:329–341. doi: 10.1093/heapol/14.4.329.
2. Mills A, Ataguba JE, Akazili J, Borghi J, Garshong B, Makawia S, Mtei G, HarrisB, Macha J, Meheus F and McIntyre D. (2012) Equity in financing and use of health care in Ghana, South Africa, and Tanzania: Implications for paths to universal coverage. Lancet. 14; 380 (9837): 126-33.
3. Nguyen HT, Rajkotia Y and Wang H. (2011) The financial protection effect of Ghana National Health Insurance Scheme: evidence from a study in two rural districts. International Journal of Equity Health 19; 10: 4.
4. Mensah J, Oppong JR and Schmidt CM. (2010) Ghana's National Health Insurance Scheme in the context of the health MDGs: an empirical evaluation using propensity score matching. Health Economics19 S: 95-106.
5. Ghana Health Service (2005) Community-Based Health Planning And Services (CHPS). The Operational Policy (2005) Ghana Health Service Policy Document No.20.
6. Framework for the implementation of the Ouagadougou Declaration on Primary Health Care and Health Systems in Africa (2009) World Health Organization Regional Office For Africa.
7. Awoonor-Williams JK, Sory EK, Nyonator FK, Phillips JF, Wang C and Schmitt ML. (2013) Lessons from scale up of a community-based health program in Ghana Global Health: Science and Practice: 1(1) Available: [www.ghspjournal.org](http://www.ghspjournal.org). Accessed 20/10/2013
8. Binka FN, Bawah AA, Phillips JF, Hodgson A, Adjuik M and MacLeod BB. (2007) Rapid achievement of the child survival millennium development goal: evidence from the Navrongo experiment in Northern Ghana. Tropical Medicine and International Health.12 (5): 578-593.
9. Ghana Statistical Service (GSS), Health Research Unit, Ministry of Health, and ORC Macro. (2003) Ghana Service Provision Assessment Survey 2002. Calverton, Maryland: Ghana Statistical Service and ORC Macro.
10. Ahwoi K (2013) Government Decentralization Policy Plan (unpublished presentation). Presented at Ghana Health Service Second Senior Managers’ Meeting Miklin Hotel-Accra.
11. Ministry of Health Ghana (1997). Medium-Term Health Strategy (1997 – 2001). Ministry of health.
12. WHO. 2007*. Everybody’s Business: Strengthening Health Systems to Improve Health Outcomes: WHO’s Framework for Action,* Geneva: WHO.
13. Report on the National Health Accounts Ghana 2005
14. Report on the National Health Accounts Ghana 2010
15. Debpuur C, Phillips JF, Jackson JF, Nazzar AK, Ngom P, and Binka FN (2002) “The impact of the Navrongo Project on contraceptive knowledge and use, reproductive preferences, and fertility.” *Studies in Family Planning* 33: 141-163.
16. Awoonor-Williams JK, Bawah [AA](http://www.biomedcentral.com/1472-6963/13/S2/S3/#ins2), Nyonator [FK](http://www.biomedcentral.com/1472-6963/13/S2/S3/#ins3), Asuru [R](http://www.biomedcentral.com/1472-6963/13/S2/S3/#ins4), Oduro [A](http://www.biomedcentral.com/1472-6963/13/S2/S3/#ins5), Ofosu [A](http://www.biomedcentral.com/1472-6963/13/S2/S3/#ins6) and Phillips JF (2013). The Ghana essential health interventions program: a plausibility trial of the impact of health systems strengthening on maternal & child survival. BMC Health Services Research. 13(S2): S3. Available at: http://www.biomedcentral.com/1472-6963/13/S2/S3
17. Nyonator, F. (2013) Universal Health Coverage: Extending Population Coverage in a Low Income Setting: The Experience of Ghana. (Unpublished) Abstract presentation at TICAD 5 August 2013.
18. Abrebese, J. (2011) Social Protection in Ghana. An overview of existing programmes and their prospects and challenges. Available:

<http://www.fesghana.org/uploads/PDF/FES_SocialProtectionGhana_2011_FINAL.pdf>

1. Ghana Health Service (2012) Ghana Health Service 2012 Annual Report.
2. Ghana Health Service (2012) Rapid Assessment of Health Insurance Capitation in Ashanti Region (unpublished report) Powerpoint presentation at Ministry of Health 2013 Health Summit. GIMPA
3. National Health Insurance Authority 2012 Annual Report
4. Jehu-Appiah C, Aryeetey G, Spaana E, de Hoop T, Agyepong I and Baltussena R (2011). Equity aspects of the National Health Insurance Scheme in Ghana: Who is enrolling, who is not and why? Social Science & Medicine 72 (2011) 157e165
5. Sarpong N, Loag W, Fobil J, Meyer CG, Adu-Sarkodie Y, May J and Schwarz NG (2010), National health insurance coverage and socio-economic status in a rural district of Ghana. Tropical Medicine & International Health, 15: 191–197. doi: 10.1111/j.1365-3156.2009.02439.x
6. Ghana Health Service District Health Information Management System2. Available: [www.ghsdhims.org](http://www.ghsdhims.org).
7. Ministry of Health. 2012 Holistic Assessment of the Health Sector. Available at: http://www.mohghana.org/UploadFiles/Publications/2012%20Holistic%20Assessment%20Report%20ofoe130715062103.pdf
8. Ghana Statistical Service (GSS), Noguchi Memorial Institute for Medical Research (NMIMR), and ORC Macro (2004) Ghana Demographic and Health Survey 2003, Calverton, Maryland: GSS, NMIMR, and ORC Macro.
9. Demographic and Health Survey. Calverton, Maryland, USA: ICF Macro. Ghana Statistical Service (GSS), Ghana Health Service (GHS), and ICF Macro (2009) Ghana Demographic and Health Survey 2008. Accra, Ghana: GSS, GHS, and ICF Macro.
10. Government of Ghana, Ghana Statistical Service, Ghana Health Service, Accra, Ghana, UNICEF, UNFPA, Japan Official Development Assistance, USAID and ICF International, Calverton, Maryland, USA (2012) Multiple Indicator Cluster Survey 2011.
11. Gupta N, Maliqi B, França A, Nyonator F, Pate MA, Sanders D, Belhadj H, Daelmans B (2011) [Human resources for maternal, newborn and child health: from measurement and planning to performance for improved health outcomes](http://www.human-resources-health.com/content/9/1/16), Human Resources for Health 2011. 9:16
12. Campbell J, Buchan J, Cometto G, David B, Dussault G, Fogstad H, Fronteira I, Lozano R, Nyonator F, Pablos-Méndez R, Quain EE, Starrs A and Tangcharoensathien V (2013) Human resources for health and universal health coverage: fostering equity and effective coverage. Available: <http://www.who.int/bulletin/volumes/91/11/13-118729.pdf>. Accessed: 10/11/2013
13. Ghana Statistical Service and Macro International Inc. (1994) Ghana Demographic and Health Survey 1993. Macro International Inc. Calverton, Maryland, USA
14. National Population Council (2011) Ghana Population Stabilization Report. Available: <http://www.populationcommunication.com/Medias/Ghana_report.pdf> Accessed: 9/11/2013
15. Ghana Statistical Service (2012) 2010 Population and Housing Census. Ghana Statistical Service
16. Biritwum R. Ghana - Study on Global Ageing and Adult Health-2007/8, Wave 1 Study on Global Ageing and Adult Health. WHO Multi-Country Studies Data Archive.
17. World Bank Report (2009) Table 2.1: Ghana’s progress toward the Millennium Development Goals. World Bank.

| \|  \| \| Type of indicator \| \| Dimension of UHC being measured \| Usefulness for monitoring UHC \| Source \| \| --- \| --- \| --- \| --- \| --- \| --- \| --- \| \| **Equity: Under-five mortality ratio- Ratio of first wealth quintile to fifth wealth quintile** \| \| Equity \| \| Population coverage \| Useful \| DHS/MICS \| \| **Equity: Geography - Supervised deliveries- Ratio of best performing region to worst performing region** \| \| Equity \| \| Service coverage \| Useful \| Routine Health service data \| \| **Equity: Geography - Ratio of best performing region to worst performing region (nurse: population ratio)** \| \| Equity \| \| Access to service \| Useful \| Human Resource - Ministry of health \| \| **Equity: NHIS - Gender (Female/Male cardholder ratio)** \| \| Equity \| \| Financial Risk protection \| Useful \| NHIA \| \| **Equity: NHIS - (Ratio of Fifth wealth quintile to the first wealth quintile who holds NHIS cards)** \| \| Equity \| \| Financial Risk protection \| Useful \| NHIA \| \| **Outpatients attendance per capita (OPD)** \| \| Coverage \| \| Population coverage \| Useful \| Routine Health service data \| \| **Doctor: population ratio** \| \| Input \| \| Access to service \| Useful- if compared across regions \| Human Resource - Ministry of health \| \| **Midwife: WIFA ratio** \| \| Input \| \| Access to service \| Useful- if compared across regions \| Human Resource - Ministry of health \| \| **Number of functional CHPS zones** \| \| Coverage \| \| Access to service \| Useful \| Routine Health service data \| \| **Nurse: population ratio** \| \| Input \| \| Access to service \| Useful if compared across regions \| Human Resource - Ministry of health \| \|  \|  \| \| \| \| \| \| \| **% Total MTEF allocation on health** \| \| Financial protection \| \| Financial Risk protection \| Useful \| MOH \| \| **% Non-wage GOG recurrent budget allocated to district level and below** \| \| Financial protection \| \| Financial Risk protection \| Useful \| MOH \| \| **Per capita expenditure on health** \| \| Financial protection \| \| Financial Risk protection \| Useful \| MOH \| \| **Budget execution rate (Item 3**  **or service as proxy)** \| \| Financial protection \| \| Financial Risk protection \| Not useful \| MOH \| \| **% of annual budge allocations to items 2 and 3 (GOG and SBS) disbursed to BMCs by end of year** \| \| Financial protection \| \| Financial Risk protection \| Useful \| MOH \| \| **% of population with valid NHIS membership card** \| \| Financial protection \| \| Financial Risk protection \| Useful \| NHIA \| \| **Proportion of claims settled within 12 weeks** \| \| Financial protection \| \| Financial Risk protection \| Not useful \| NHIA \| \| **% Internally Generated Funds from NHIS** \| \| Financial protection \| \| Financial Risk protection \| Not useful \| MOH \| \| **Maternal Mortality Ratio (MMR) per 100,000 live births** \| \| Impact \| \| Population coverage \| Useful \| Maternal Health Survey \| \| **Total Fertility Rate** \| \| Impact \| \| Population coverage \| Useful \| MICS/DHS \| \| **Family planning coverage** \| \| Coverage \| \| Population coverage \| Useful \| GHS \| \| **% Of pregnant women attending at least 4 antenatal visits** \| \| Coverage \| \| Service coverage \| Useful \| Routine Health service data \| \| **Infant Mortality Rate (IMR) per 1,000 live births** \| \| Impact \| \| Population coverage \| Useful \| MICS/DHS \| \| **Under 5 Mortality Rate (U5MR) per 1,000 live births** \| \| Impact \| \| Population coverage \| Useful \| MICS/DHS \| \| **% Deliveries attended by a trained health worker** \| \| Coverage \| \| Service coverage \| Useful \| Routine Health service data \| \| **% Children 0-6 months exclusive breastfed** \| \| Impact \| \| Population coverage \| Useful \| MICS/DHS \| \| **Under 5 prevalence of low weight for age** \| \| Impact \| \| Population coverage \| Useful \| MICS/DHS \| \|  \|  \| \| \| \| \| \| \| **HIV prevalence among pregnant women 15-24 years** \| \| \| Impact \| Population coverage \| Not useful \| NACP \| \| **% of U5s sleeping under ITN** \| \| \| Coverage \| Population coverage \| Useful \| MICS/DHS \| \| **% of children fully immunized by age one – Penta-3** \| \| \| Coverage \| Service coverage \| Useful \| Routine Health service data \| \| **HIV+ clients ARV treatment** \| \| \| Coverage \| Service coverage \| Useful \| NACP \| \| **Incidence of Guinea Worm** \| \| \| Coverage \| Service coverage \| Useful \| Routine Health service data \| \| % Households with improved sanitary facilities \| \| \| Coverage \| Population coverage \|  \| MICS/DHS \| \| % Households with access to improved source of drinking water \| \| \| Coverage \| Population coverage \| Useful \| MICS/DHS \| \| Obesity in population (women aged 15-49 years) \| \| \| Coverage \| Population coverage \| Useful \| DHS \| \| TB treatment success rate \| \| \| Coverage \| Service coverage \| Useful \| NTP \| \| Equity index: Ratio of mental health nurses to patient population \| \| \| Equity \| Access \| Useful \| Human Resource - Ministry of health \| \| Number of community psychiatric nurses trained and deployed \| \| \| Output \| Access \| Useful if compared across regions \| Human Resource - Ministry of health \| \| % Tracer psychotropic drug availability in hospitals \| \| \| Impact \| Access \| Useful \| Routine Health service data \| \| Institutional infant mortality rate \| \| \| Impact \| Service coverage \| Useful, but biased \| Routine Health service data \| \| Basket equipment functioning in hospitals \| \| \| Coverage \| Service coverage \|  \| Facility Survey \| \| % Tracer drugs availability in hospitals \| \| \| Impact \| Access \| Useful \| Routine Health service data \| \| % of hospitals assessed for quality assurance and control \| \| \| Coverage \| Service coverage \| Useful \| Facility Survey \| \| Institutional under-five mortality rate \| \| \| Impact \| Service coverage \| Useful \| Routine Health service data \| \| Institutional MMR \| \| \| Impact \| Service coverage \| Useful \| Routine Health service data \|  1. **Table S3**: Sectorwide Indicators [25] |
| --- | --- | --- | --- | --- | --- | --- | --- | --- | --- | --- | --- | --- | --- | --- | --- | --- | --- | --- | --- | --- | --- | --- | --- | --- | --- | --- | --- | --- | --- | --- | --- | --- | --- | --- | --- | --- | --- | --- | --- | --- | --- | --- | --- | --- | --- | --- | --- | --- | --- | --- | --- | --- | --- | --- | --- | --- | --- | --- | --- | --- | --- | --- | --- | --- | --- | --- | --- | --- | --- | --- | --- | --- | --- | --- | --- | --- | --- | --- | --- | --- | --- | --- | --- | --- | --- | --- | --- | --- | --- | --- | --- | --- | --- | --- | --- | --- | --- | --- | --- | --- | --- | --- | --- | --- | --- | --- | --- | --- | --- | --- | --- | --- | --- | --- | --- | --- | --- | --- | --- | --- | --- | --- | --- | --- | --- | --- | --- | --- | --- | --- | --- | --- | --- | --- | --- | --- | --- | --- | --- | --- | --- | --- | --- | --- | --- | --- | --- | --- | --- | --- | --- | --- | --- | --- | --- | --- | --- | --- | --- | --- | --- | --- | --- | --- | --- | --- | --- | --- | --- | --- | --- | --- | --- | --- | --- | --- | --- | --- | --- | --- | --- | --- | --- | --- | --- | --- | --- | --- | --- | --- | --- | --- | --- | --- | --- | --- | --- | --- | --- | --- | --- | --- | --- | --- | --- | --- | --- | --- | --- | --- | --- | --- | --- | --- | --- | --- | --- | --- | --- | --- | --- | --- | --- | --- | --- | --- | --- | --- | --- | --- | --- | --- | --- | --- | --- | --- | --- | --- | --- | --- | --- | --- | --- | --- | --- | --- | --- | --- | --- | --- | --- | --- | --- | --- | --- | --- | --- | --- | --- | --- | --- | --- | --- | --- | --- | --- | --- | --- | --- | --- | --- | --- | --- | --- | --- | --- | --- | --- | --- | --- | --- | --- | --- | --- | --- | --- | --- | --- | --- | --- | --- | --- | --- | --- | --- | --- | --- | --- | --- | --- | --- | --- | --- | --- | --- | --- | --- | --- | --- | --- | --- | --- | --- | --- | --- | --- | --- | --- | --- | --- | --- | --- | --- | --- | --- | --- | --- | --- | --- | --- | --- | --- | --- | --- | --- | --- |
